# Study of the correlation between sensing performance and surface morphology of inkjet-printed aqueous graphene-based chemiresistors for NO_2_ detection

**DOI:** 10.3762/bjnano.8.103

**Published:** 2017-05-09

**Authors:** F Villani, C Schiattarella, T Polichetti, R Di Capua, F Loffredo, B Alfano, M L Miglietta, E Massera, L Verdoliva, G Di Francia

**Affiliations:** 1 ENEA - R.C. Portici, Piazzale E. Fermi 1, I-80055, Portici (Naples), Italy; 2 Dipartimento di Fisica "E. Pancini", Università di Napoli "Federico II", Via Cintia, I-80126, Naples, Italy; 3 CNR-SPIN UOS Napoli, Via Cintia, I-80126, Naples, Italy

**Keywords:** aqueous graphene dispersion, gas sensors, inkjet printing, liquid phase exfoliation, nitrogen dioxide, paper-based electronics

## Abstract

The extremely high sensitivity to the external environment and the high specific surface area, as well as the absence of bulk phenomena that could interfere with the response signal, make graphene highly attractive for the applications in the field of sensing. Among the various methods for producing graphene over large areas, liquid phase exfoliation (LPE) appears to be very promising, especially if combined with inkjet printing (IJP), which offers several advantages, including the selective and controlled deposition of small ink volumes and the versatility of the exploitable inks and substrates. Herein we present a feasibility study of chemiresistive gas sensors inkjet-printed onto paper substrates, in which a LPE graphene suspension dispersed in a water/isopropanol (H_2_O/IPA) mixture is used as sensing ink. The device performances, in terms of relative conductance variations, upon exposure to NO_2_ at standard ambient temperature and pressure, are analysed. In addition, we examine the effect of the substrate morphology and, more specifically, of the ink/substrate interaction on the device performances, by comparing the response of different chemiresistors fabricated by dispensing the same suspension also onto Al_2_O_3_ and Si/SiO_2_ substrates and carrying out a supportive atomic force microscopy analysis. The results prove the possibility to produce sensor devices by means of a wholly environmentally friendly, low-cost process that meets the requests coming from the increasing field of paper-based electronics and paving the way towards a flexible, green-by-design mass production.

## Introduction

In the recent years, the potential of graphene for sensing applications has been largely explored because of its outstanding properties [1]: extremely high sensitivity to the external environment, excellent surface-to-volume ratio, as well as the absence of bulk phenomena that interfere with the response signal. All these peculiarities of graphene are widely attracting the interest of the scientific community involved in this research field, and much attention is devoted to the fabrication methods. Liquid phase exfoliation (LPE) [2] is a very promising fabrication technique among different methods for producing graphene over large areas [3–4], and especially in comparison with mechanical exfoliation methods that provide high-quality material but only low throughput. LPE consists of the exfoliation of graphite in organic electron-donor solvents [5–7], which are generally high-boiling and need to be handled with care because of their toxicity. Recently, the potential to effectively exfoliate natural graphite in a hydro-alcoholic mixture in a process of low environmental impact has been demonstrated [8]. In general, the liquid-phase preparation of graphene extends its application to solution-processable deposition methods. These methods are more oriented towards large-scale production, such as printing technologies that stand out as high-throughput, low-temperature processes, also employable in roll-to-roll configuration. Among these technologies, inkjet printing (IJP) is a sustainable methodology due to a reduced number of processing steps and a minimized amount of waste materials. Indeed, IJP utilizes very minute amounts of materials, deposits selectively and patterns them at the same time under no-contact and no-vacuum conditions by means of digital masters realized by CAD software. This guarantees high precision and accuracy in the deposition process [9–10]. All these features make IJP completely versatile in terms of employable inks and substrates, the latter being either rigid or flexible. In our previous work, we have demonstrated the reliability of IJP in depositing LPE graphene-based ink in a controlled manner so as to produce sensor devices with reproducible performances in terms of electrical responses upon gas exposures [11]. This is not guaranteed by other conventional liquid-phase processes like drop-casting [12]. Hence, a LPE graphene suspension synthesized by using eco-friendly solvents deposited onto recyclable substrates by IJP well meets the technological requirements for the production of low-environmental impact, low-cost sensing devices.

The aim of the current research is a feasibility study of chemiresistive gas sensors by inkjet printing a LPE graphene suspension dispersed in a water/isopropanol (H_2_O/IPA) mixture as sensing ink onto paper substrates. Analogous paper-based graphene sensors, relying on different fabrication processes, such as CVD (chemical vapour deposition) grown transferred graphene [13] or inkjet-printed GO (graphene oxide) dispersion [14], are reported in literature. The former utilizes a highly energy-consuming method [3,13], the latter generally employs highly dangerous chemicals [4,14]. Therefore both approaches are not suitable for sustainable processes. In this study, the electrical responses of the chemiresistor have been analysed upon NO_2_ exposure at standard ambient temperature and pressure. Moreover, as comparison, inkjet-printed sensors have been manufactured on standard insulating substrates, namely alumina (Al_2_O_3_), and silicon dioxide (Si/SiO_2_). They have been characterized through gas sensing and atomic force microscopy (AFM) analyses, in order to investigate the effect of the substrate morphology and, more specifically, of the ink–substrate interaction on the device performances.

The reported analyses demonstrate the possibility to inkjet-print an aqueous graphene suspension onto flexible, environmentally friendly, low-cost substrates addressing the requirements of paper-based electronics for a cost-efficient, high-output manufacturing and opening the route towards the flexible, eco-designed mass production.

## Results and Discussion

An aqueous graphene-based dispersion has been formulated via a sonication-assisted liquid phase exfoliation of graphite in a mixture of water and isopropanol as described in details in the Experimental section and fully investigated in [8]. As already reported, the dispersed material consists of exfoliated flakes having less than five layers and a lateral size around 500 nm [8]. The concentration of the as-prepared few-layer graphene suspension, estimated through UV–vis spectroscopy, is 0.09 mg/mL (details about the UV–vis calibration curve are provided in Figure S1 of Supporting Information File 1). This value is expected because liquid-phase exfoliation processes of graphite usually lead to yields of few-layer graphene in suspension of around 10%. This can be further improved by complex recycling procedures [5,15]. However, as the printing process would benefit from the use of inks with higher concentrations of the active material, the suspension has been enriched up to the final concentration of 0.2 mg/mL (see the procedure described in the Experimental section).

The physico-chemical properties of this final formulation have been investigated in order to establish if it satisfies the physical and rheological requirements of the fluid flow in the inkjet-printing process and to determine its jettability as ink. Surface tension, dispersion stability and aggregate size have been measured since the ink printability depends on these main parameters, which have to match the operating parameters of the employed printing system.

**Figure 1:**
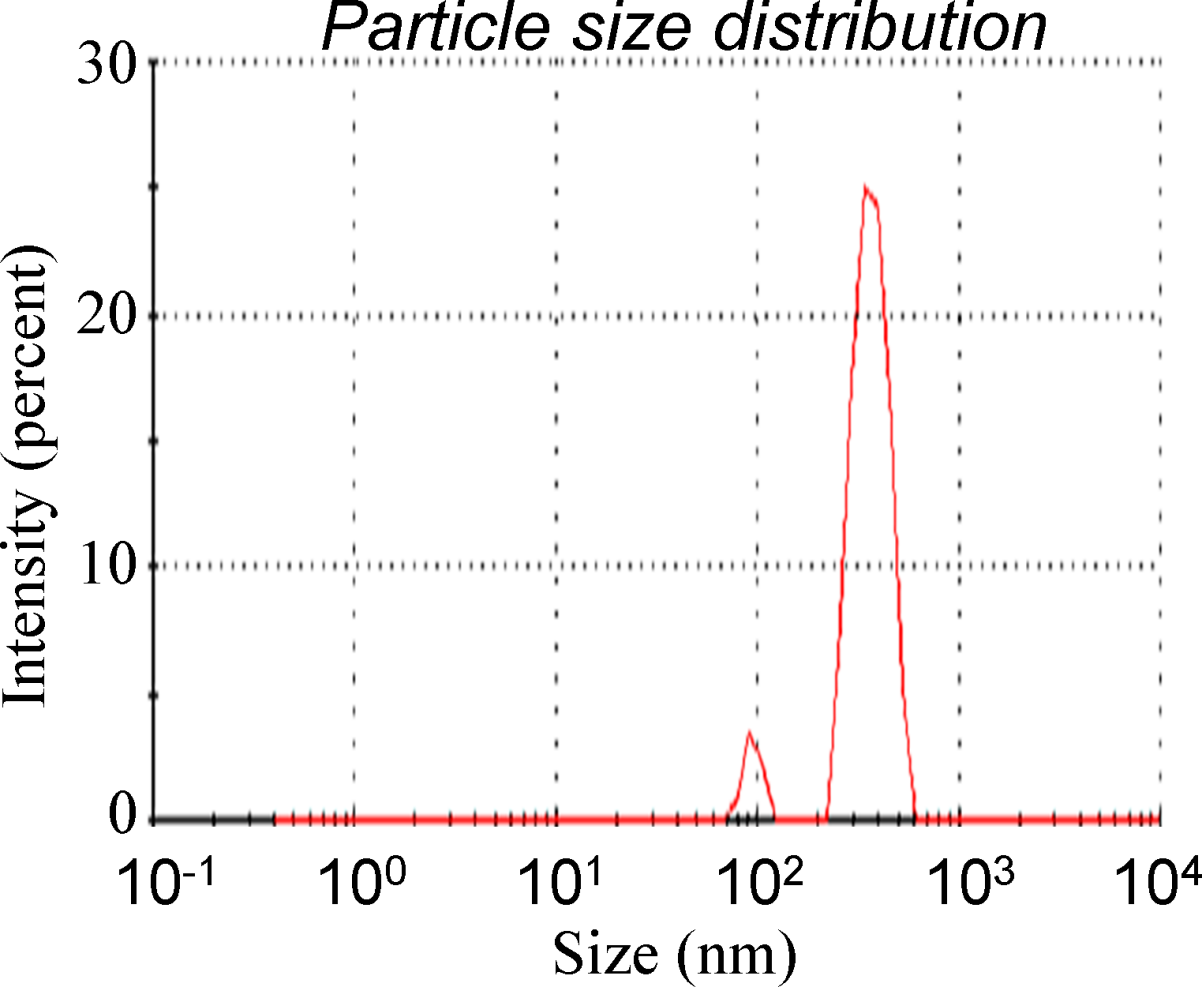
Particle size distribution of the synthesized dispersed graphene in the prepared suspension measured by DLS.

Based on these considerations, the LPE graphene dispersion has been investigated by dynamic light scattering (DLS) to determine the size distribution profile of the suspended graphene flakes. An almost monomodal distribution characterized by a low polydispersity has been detected, so indicating the homogeneity of the sample. The average size distribution profile is displayed in Figure 1. A size of *Z*_average_ = 380 ± 10 nm and a polydispersity index of 0.32 ± 0.04 have been estimated from an average over five measurements. The detected submicrometer size represents an adequate condition to avoid any clogging of the printheads. Additionally, by monitoring the dispersion through the DLS analysis, its time-stability has been confirmed over two weeks. The surface tension has been measured and the obtained value is 26.30 mN/m, which falls into the operating range (20–40 mN/m) of the printhead of the inkjet system. As a result, the performed analyses point out that the physical parameters of the dispersion are suitable for the IJP technique.

**Figure 2:**
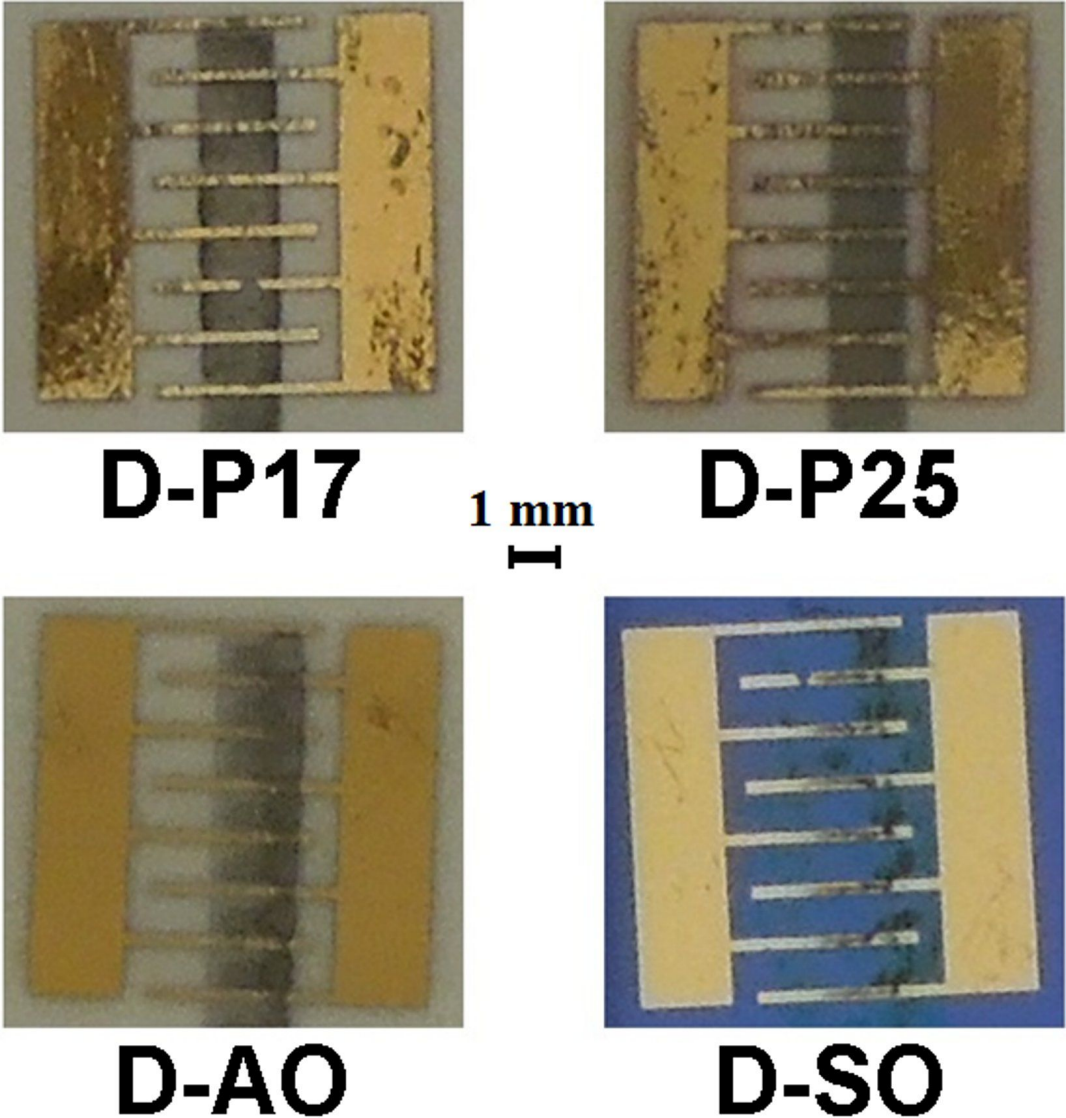
Pictures of the four investigated devices. D-P17 and D-P25 are the paper-based devices, while D-AO is the one printed on Al_2_O_3_ and D-SO is fabricated on Si/SiO_2_. The interdigitated electrodes have 8 fingers and 7 gaps; each finger is 250 μm wide and 4000 μm long, the gap between the fingers is 860 μm.

After having verified the jettability, the graphene ink has been dispensed onto paper and on two the standard insulating substrates Al_2_O_3_ and Si/SiO_2_. The devices have been manufactured according to the geometry described in the Experimental part, taking care to attain a final base resistance in the range of 1–100 kΩ. In detail, two devices have been fabricated on paper, differing for the number of printed layers, namely 17 (device D-P17, base resistance *R*_0_ = 34 kΩ) and 25 (device D-P25, *R*_0_ = 20 kΩ). The device printed on alumina (device D-AO) resulted in 31 overlapped layers, so driving the base resistance to 30 kΩ, similar to those of the paper-based devices. The fourth typology of device has been fabricated on silicon dioxide. It should be pointed out that the wetting of the aqueous graphene ink-Si/SiO_2_ system was very poor inducing a not continuous and not controlled deposition. This is expected since the measured surface energy of the substrate (30.95 mN/m) is comparable with the surface tension of the solution (26.30 mN/m). Hence, an UV–ozone treatment of the substrate was necessary to increase its surface energy, allowing for a more uniform deposition of the material. Nonetheless, the macroscopic aspect of the film printed on the treated substrate looks different from the others, with the material distributed over a larger area (Figure 2). In addition, the Si/SiO_2_-based device has not reached exactly the same base resistance value of the other two samples because the drying process redistributes the graphitic material differently, depending on the surface energy but primarily on the roughness of the substrate. All that said, the characterized device (D-SO) has been fabricated printing 38 layers, with base resistance *R*_0_ = 15 kΩ. Pictures of the four devices is displayed in Figure 2.

**Figure 3:**
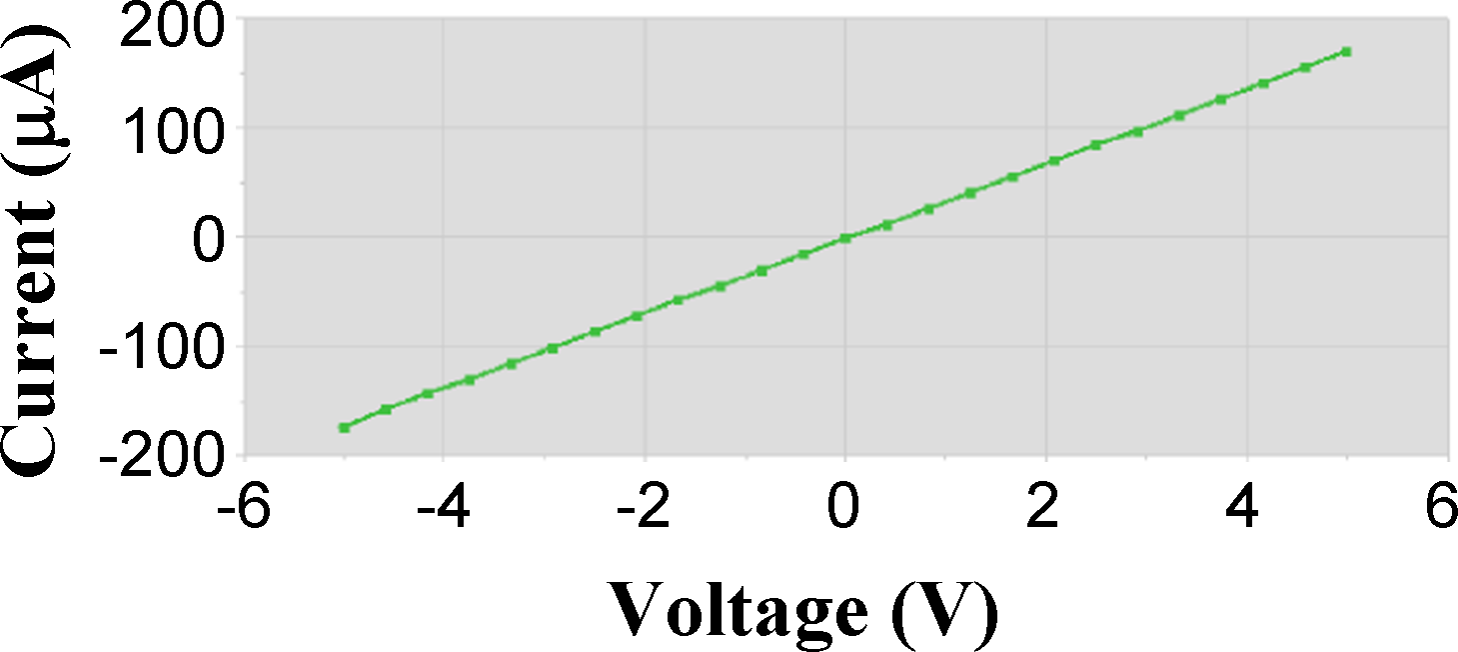
*I*–*V* curve of a chemiresistor printed on paper (D-P17). Data are collected in the range [−5 V, 5 V].

The voltamperometric characteristics of all the fabricated devices show a linear feature, confirming the formation of an ohmic contact between graphene and gold electrodes. As an example, the *I*–*V* curve of the device D-P17 is reported in Figure 3.

**Figure 4:**
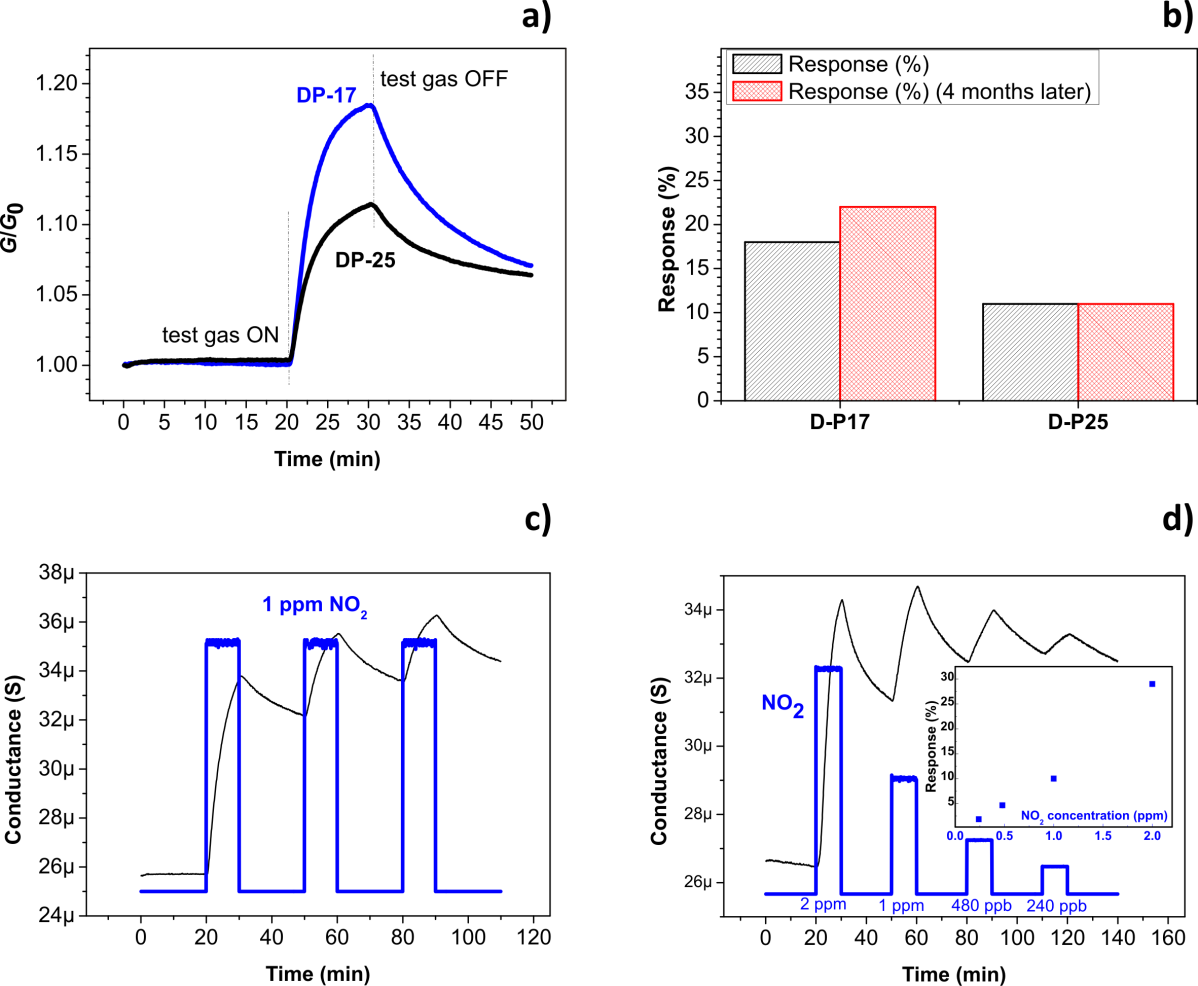
a) Dynamic responses of the paper-based devices (D-P17 blue line and D-P25 black line), exposed to 1 ppm NO_2_. The curves have been normalized to their base conductance value. b) Responses of the devices after four months. c) Response of D-P17 to three consecutive exposures to 1 ppm of NO_2_. d) Response of D-P17 to a sequence of NO_2_ injections at different concentrations. The inset shows the related sensitivity curve.

The sensing properties of the chemiresistors have been tested by exposing the devices to NO_2_, the analyte towards which LPE graphene is more specific [16]. In Figure 4a, the dynamic responses toward 1 ppm of NO_2_ for paper-based devices are reported. After an exposure of ten minutes, the devices reach a different percentage increase of the conductance *G*, defined as:

[1]
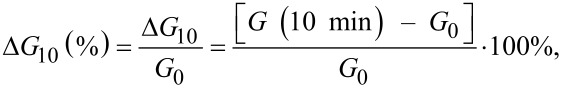


with *G*_0_ being the base conductance, measured immediately before the NO_2_ exposure. It is worth noting that the device fabricated with a lower number of printed layers (D-P17) shows a remarkably higher response (Δ*G*_10_ = 18%) respect to the device based on a thicker film (i.e., D-P25, Δ*G*_10_ = 10%) and even after several months of storage in air the same values were measured (see Figure 4b). As a first step, we could ascribe this higher sensitivity to the smaller thickness of the IJP layer in D-P17 with respect to D-P25 and thus to the higher surface-to-volume ratio of the graphene film in D-P17. In addition, the thickness of the film can have repercussions also on its morphology, as deeply analysed afterwards by AFM analysis, since a thinner layer retraces more accurately the substrate surface, so envisaging an indirect effect of the substrate onto the sensing layer morphology.

Typical for graphene-based chemiresistors operating at room temperature, the sensing curves show slow recovery behavior. This implies several hours for reaching the initial conductance values or the recourse to UV or heating treatments [17]. This is still a limitation of the devices, especially when the devices are exposed to subsequent cycles of exposures, as shown in Figure 4c,d, which display the dynamic behavior of D-P17 (the best-performing paper-based device). Figure 4c shows three subsequent exposures to 1 ppm of NO_2_. In this test, the analyte flows for 10 min, then the device is purged for 20 min with carrier gas and the procedure is repeated three times. As a consequence of slow recovery the device was not in its “zero state” at the beginning of the second exposure. The recorded Δ*G*_10_ value was lower (about 9%), and even lower (about 6%) in the third step. In Figure 4d we report the response of D-P17 to a sequence of NO_2_ injections at different concentrations (in the range of 0.24–2 ppm). The measured Δ*G*_10_ values are reported as a function of the concentration in the inset. Both the dynamic behavior and the plot “response vs concentration” demonstrate how the device is still clearly sensitive to 0.24 ppm NO_2_, even starting very far from its “zero state”. Anyway, as elsewhere reported, the highlighted issues related to the slow recovery could be circumvented by recurring to suitable analyses of the sensing signal [2,18].

**Figure 5:**
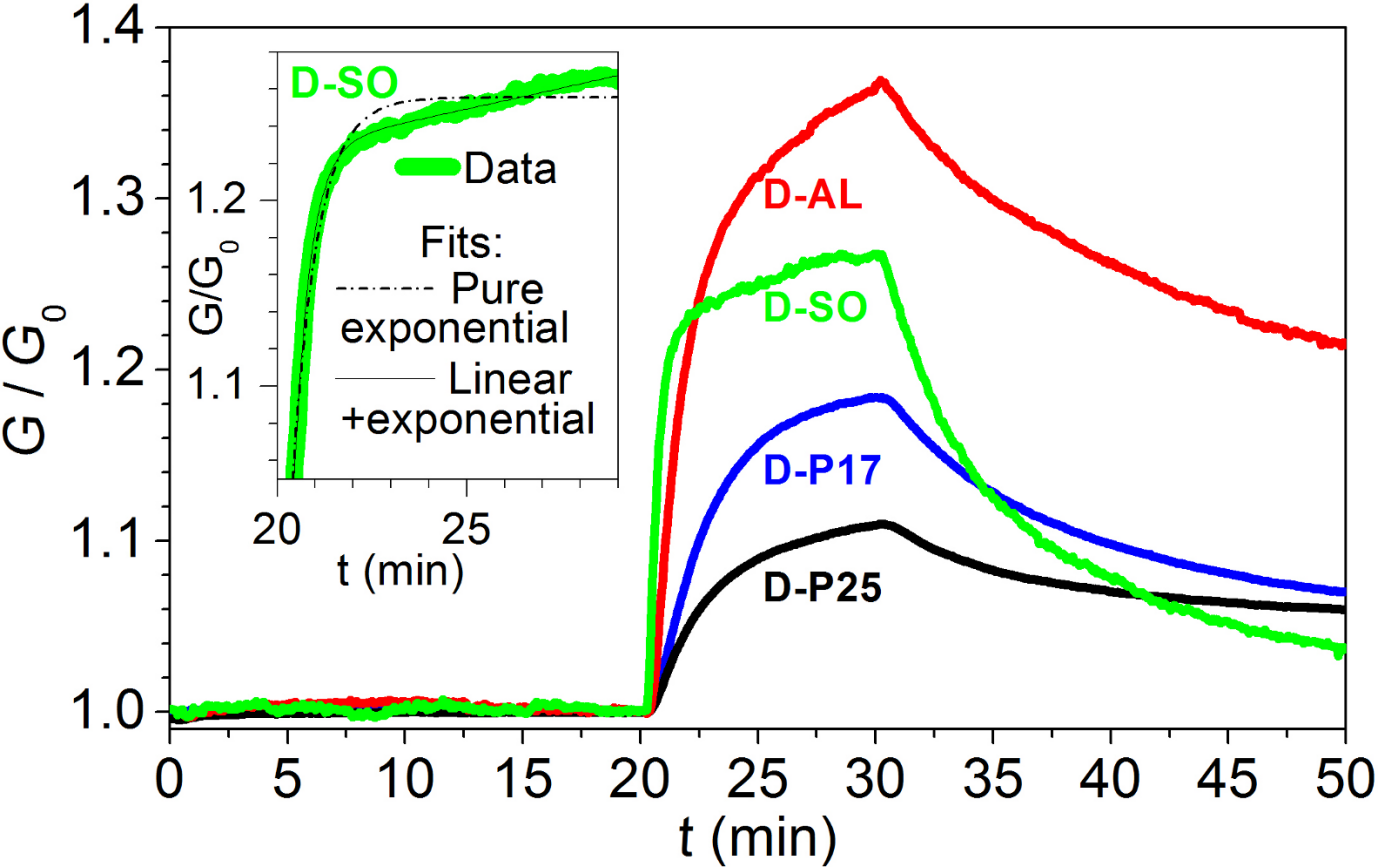
Dynamic responses of the four investigated devices exposed to 1 ppm NO_2_. The curves have been normalized to their base conductance value. In the inset the magnification of the rising *G* for device D-SO and two related fit curves are shown. One fit (dotted line) is calculated with a pure exponential function, whereas the other (solid line) comes from a sum of an exponential function and a linear term.

Paper substrates represent a quite hot topic in the field of chemical sensing. In order to better understand how and how much the choice of the paper as substrate influences the performances of printed graphene sensors, a comparison with devices obtained on conventional substrates has been done. To this aim, the same graphene-based ink has been deposited by IJP on Al_2_O_3_ and SiO_2_, and the sensing performances of devices obtained with these conventional substrates were compared with those of paper-based devices. All chemiresistors have been exposed according to the same protocol (Figure 5).

Despite the fact that all the devices are constituted of the same sensing material, they exhibit quite different values of relative response to the analyte (Table 1). The layers printed onto the conventional substrates showed faster responses and higher sensitivities towards NO_2_. The better performances of these devices are, however, contrasted by a lack of stability. After storage in air for four months, D-P17 and D-P25 preserved their properties (Figure 4b), while D-AO and D-SO extremely degraded (Δ*G*_10_ decreased to less than a fourth for both devices). This behavior is rather unexpected and it deserves to be further investigated in a future work since, according to our experience, devices prepared with the same type of graphene dispersion onto Al_2_O_3_ and Si/SiO_2_ by drop-casting are stable and show better sensing performances over much longer times [16].

**Table 1:** Main features and estimated properties of the investigated devices.

device	substrate	number of inkjet-printed layers	*R*_0_ (kΩ)	Δ*G*_10_	τ (s)	*A* (nS/s)

D-P17	paper	17	34	18.4%	142	0.2
D-P25	paper	25	20	10.9%	118	1.5
D-AO	Al_2_O_3_	31	30	36.6%	97	1.9
D-SO	Si/SiO_2_	38	15	26.8%	25	5.4

In order to better understand the different sensing behavior, and possibly to obtain information about the underlying microscopic mechanisms, we have studied the time evolution of the conductance, *G*(*t*), during the gas exposure. On D-P17, *G*(*t*) can be well fitted by the following law:

[2]
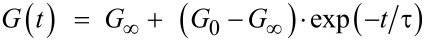


representing an exponential increase of *G*, with a characteristic time constant τ, from the initial value *G*_0_ to the asymptotic value *G*_∞_. The above written law, however, fails to satisfyingly fit *G*(*t*) on the other devices. This is particularly true for D-SO on which to the initial exponential increase follows further increase that is nearly linear. This suggests that the most appropriate function for *G*(*t*) should include an additional linear term to the previous exponential form:

[3]
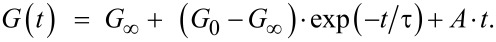


This function yields a more suitable fit for *G*(*t*) over the whole time range of the increase of *G* for all the devices. As an example, in the inset of Figure 5 we show a section of the increase of *G* of D-SO during the exposure to NO_2_, together with the best fit curves obtained with the two illustrated choices of *G*(*t*). It is easy to note how in this case the pure exponential fit fails and how a linear term must be added. Table 1 summarizes the basic features of the realized devices and the main measured parameters together with the values of the fitting parameters for all devices. As expected for D-P17 where the exponential law was already satisfying, the *A* parameter is almost zero. On the contrary, the exponential term exhibits the highest τ value, which corresponds to the slowest increase of the conductance signal followed in sequence by D-P25, D-AO and D-SO, as observed from the curves in Figure 5.

The overall electrical features and the detected differences in the sensing behaviors of the devices on different substrates have been further investigated, in order to understand whether there is any correlation with the surface features of the printed material. To this end, a morphological investigation with AFM has been performed.

**Figure 6:**
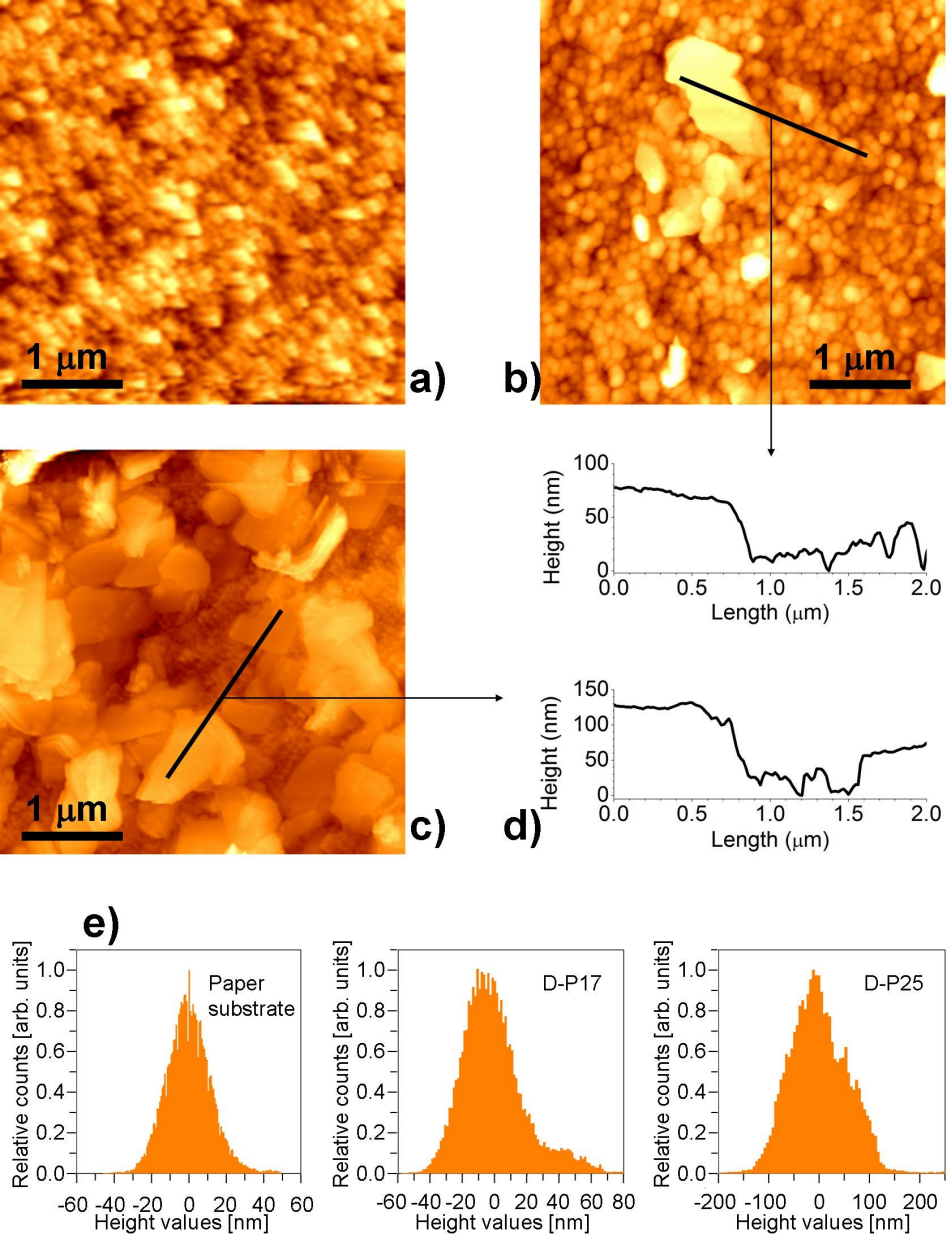
a) AFM image of the paper substrate (rms roughness: 12 nm). b,c) Typical AFM images on LPE graphene printed on paper (the images have been recorded on D-P17 (rms roughness: 21 nm) and D-P25 (rms roughness: 59 nm), respectively). d) Height profiles along the lines highlighted in the images of panels b) and c). e) Distribution of heights measured on the samples surfaces (relative counts are normalized to the peak value).

**Figure 7:**
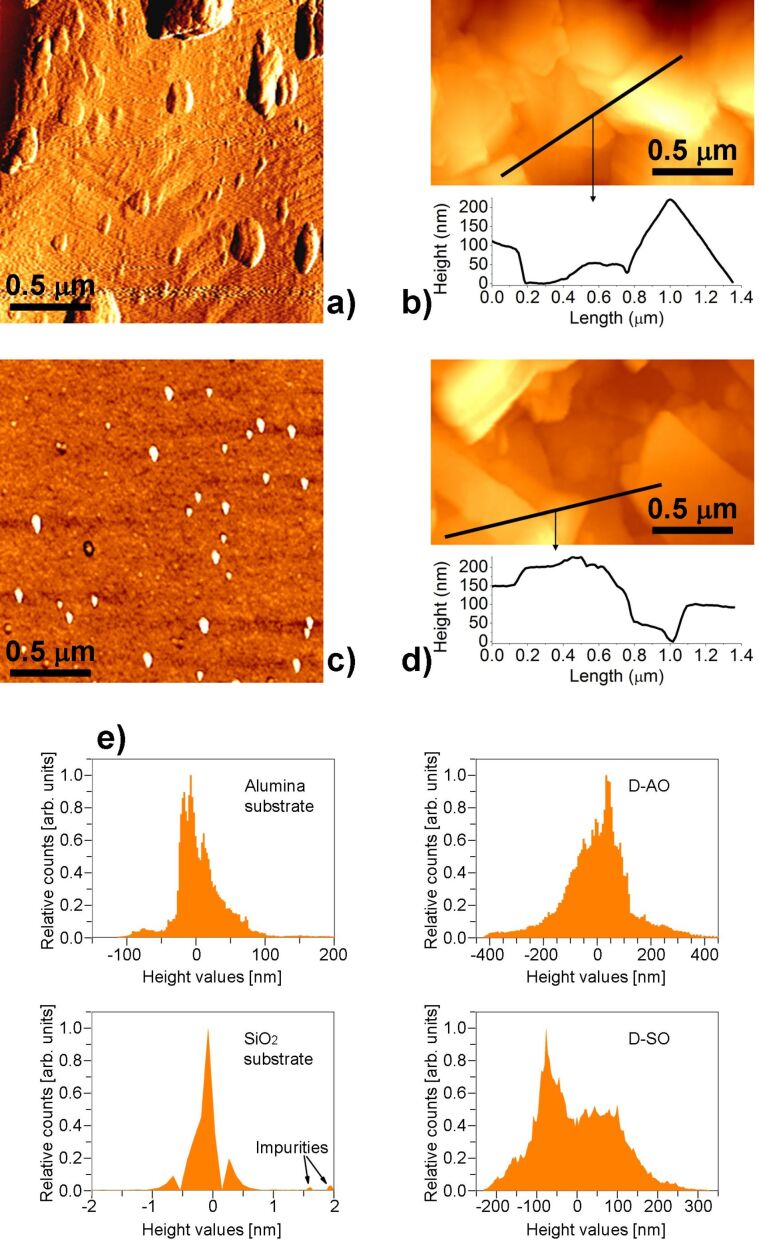
a) AFM image of the Al_2_O_3_ substrate (rms roughness: 35 nm). b) AFM image of LPE graphene printed on Al_2_O_3_ in D-AO device (rms roughness: 120 nm); a height profile crossing the regular structures imaged on the surface is also reported. c) AFM image of the Si/SiO_2_ substrate (rms roughness: 0.7 nm). d) AFM image of LPE graphene printed on Si/SiO_2_ in D-SO device (rms roughness: 96 nm), a height profile crossing the regular structures imaged on the surface is also reported. e) Distribution of heights measured on the samples surfaces (relative counts are normalized to the peak value).

Figure 6 and Figure 7 report typical AFM images of the surface morphology of the graphene material on each device, together with the morphology of each substrate. A striking difference is observed on the two devices on paper with respect to the other two ones. LPE graphene on D-P17 and D-P25 exhibits large generally granular regions (with grains having lateral sizes of the order of 100 nm). Some planar flakes are found lying on these grains, with some surface portions richer of these features than others. Sampling several images on different regions of both devices, we have found that D-P17 shows a predominantly granular morphology. Differently from these two devices, D-AO and D-SO exhibit surface morphologies characterized by randomly orientated planar flakes having submicrometer lateral size.

Concerning the recorded base resistances, a poorer electrical connection between separated planar structures than between grains could be at the origin of the higher number (nearly double) of printed layers necessary to make the conductance of D-AO similar to the one of D-P17. The anomalous behavior of D-SO can be ascribed to the observed peculiar spreading of LPE graphene on the SiO_2_ substrate: A marked material accumulation on the edge of the drop due to a significant coffee ring effect is visible. This ring constitutes an effective bulk path, which results in a lower base resistance. The edge accumulation is also responsible of the difficulties in controlling the base conductance value as a function of the number of inkjet-printed layers.

Regarding the electrical responses and their correlation to the surface morphology of the printed material, the concentration of active sites, which plays an important role in the sensing process, must be taken into account [19]. The sensing material is the same for all devices. Hence, we can assume that the concentration of active sites is nearly the same for all of them. In the case of graphene the active sites are represented by sp^2^ carbon atoms (low interaction energy) and defects such as vacancies, dangling bonds, coordination defects and functionalizations (high interaction energy). In the specific case of our LPE graphene, defects are mostly ascribed to edge defects that are not chemically modified by the interaction with the substrates (see Figure S2 in Supporting Information File 1).

A first analysis of the experimental results suggests that the clear difference in surface appearance could be the reason for the larger response (quantified by Δ*G*_10_) of D-AO and D-SO compared to the devices on paper, indicating that the planar surface of the flakes promotes larger adsorption. In this perspective, the higher value of Δ*G*_10_ for D-P17 compared with D-P25 seems to contradict the higher statistical abundance of planar structures on the latter. Such apparent discrepancy could be explained by considering the additional effect related to the sensing film thickness as previously mentioned, i.e., the higher surface-to-volume ratio of D-P17 with respect to D-P25. From these premises, we can deduce the coexistence of two different adsorption mechanisms which provide a physical meaning to the functional terms employed to model *G*(*t*): the adsorption of the analyte on the surface and the consequent diffusion of NO_2_ molecules inside the bulk, i.e., the portion of the sensing film not directly exposed to the gas.

As the time scale of the diffusion process is much longer than the measuring time, the behavior of this mechanism can be approximated by a linear function, leading to the linear term in Equation 3. The different morphology of the material, grown on various substrates, also plays an important role in the diffusion of the gas molecules through the sensing film. A layer mainly consisting of flakes rather than grains promotes greater gas diffusion inside the bulk, thanks to a larger effective volume, thereby indicating the dead-volume among the adjacent structures, which is available for the gas permeation. This can explain the higher adsorption observed on D-AO and D-SO, as well as the increase of the role of the linear term (quantified by the parameter *A*) when the amount of planar structures increases.

In addition, the adsorption probability of a single incident molecule increases when the direction of incidence approaches the normal of the local surface (because the normal component of the momentum increases) [20]. This is consistent with the larger adsorption at and the faster response from planar surfaces. Indeed, we observed that τ decreases from D-P17 to D-P25, to D-AO and D-SO. In this respect, the τ estimated on D-SO, significantly smaller than the one estimated on D-AO despite the similar surface appearance, could be not representative solely for surface features, since D-SO exposes a larger area which could imply faster exchange. This interpretation seems to be confirmed by the recovery curves after stopping the exposure to NO_2_. In this process D-SO also exhibited the fastest behavior. Once again, this agrees well with the overall picture and with the higher surface-to-volume ratio of graphene on this device.

## Conclusion

A feasibility study on the fabrication of graphene-based chemiresistors, employing sustainable technologies and materials, namely by inkjet printing a hydro-alcoholic ink on paper, has been carried out. DLS, UV–vis and surface tension characterizations showed the suitability of the LPE graphene dispersion as ink for implementing inkjet printing on paper. The influence of the substrate morphology on the sensing performance has been examined further considering two standard insulating substrates as comparison, Si/SiO_2_ and Al_2_O_3_. All devices have been tested towards 1 ppm NO_2_ and showed to work at standard ambient temperature and pressure. Atomic force microscopy allowed to probe the effect of the morphology of the printed materials onto different substrates on the gas sensing behavior. Inkjet-printed paper-based devices exhibited poorer performances with respect to those on standard substrates in terms of conductance variation. This can be ascribed to the peculiar arrangement of the deposited material. Notwithstanding, a reliability in the control of the base parameters (i.e., base resistance, sensing response and stability) has been clearly observed on paper substrate. In this frame, inkjet printing with its ability to control the thickness of the deposited ink appears a more powerful tool to device fabrication in comparison to other techniques commonly used, e.g., drop-casting deposition.

Overall, the main drawback in paper-based devices is the surface morphology of the sensing film, induced by the granular surface of the substrate. The Al_2_O_3_-based device, which shows a surface characterized by planar structures, exhibited remarkably higher response. For this reason, future perspectives are oriented towards the development of proper surface treatments to promote the planar assembling of graphene flakes onto the paper substrate. In every respect, the results indicate the possibility to produce sensor devices by means of a wholly environmentally friendly, low-cost process that meets the requests coming from the increasing field of paper-based electronics and paving the way towards a flexible, green-by-design mass production.

## Experimental

### Materials

Graphite flakes (product 332461) and isopropyl alcohol (IPA, RS for HPLC, isocratic grade) have been purchased from Sigma-Aldrich and Carlo Erba, respectively. Ultrapure water has been obtained with a Type 1 (Ultrapure) Milli-Q system. Colloidal suspensions have been prepared by mixing graphite flakes at 1 mg/mL in a solvent mixture IPA/H_2_O (1:7) and sonicating in an ultrasonic bath (output power ca. 30 W) for about 48 h. In order to avoid undesired overheating of the water bath during the prolonged sonication, the temperature of the bath has been fixed at 50 °C. The water level has been kept constant by installing a syphon system.

Afterwards, the suspension has been centrifuged for 45 min at 1000 rpm in order to remove unexfoliated graphite crystallites. The final ink has been obtained by taking 20 mL of this as-prepared graphene suspension, drying it in vacuum at 40 °C overnight, then adding 10 mL of fresh solvent mixture and briefly sonicating for 10 min.

### Characterizations

The concentration of the dispersed material soon after centrifugation has been determined by filtering a known volume of dispersion through a nylon membrane. The filter has been dried overnight in vacuum at 40 °C and the filtered mass was carefully measured, taking into account the filter mass. UV–vis (200–850 nm) transmittance spectra of a diluted set of this dispersion have been performed by means of a Shimadzu UV-1800 UV–vis spectrophotometer. In all cases, UV–vis absorbance spectra have been measured that appeared flat and featureless [5]. The absorption coefficient calculated at 660 nm trough the Lambert–Beer law has been <α_660_> = 1920 ± 13 L·g^−1^·m^−1^ (the UV–vis calibration curve is reported in Figure S1 of Supporting Information File 1). The concentration of the final ink has been then estimated through UV–vis measurements of the sample absorbance at 660 nm. The surface tension of the prepared dispersion has been evaluated by using the contact angle equipment OCA20-Dataphysics in pendant drop configuration. The measurements have been performed under ambient conditions (21 °C, RH 50%) and the estimated value has been the result of measurements repeated five times for each sample.

The same system in static sessile drop configuration has been employed to perform substrate surface energy (SE) measurements. This analysis has been carried out by using water as polar solvent and dichloromethane as nonpolar solvent and the measurement results, coming from a sampling of five measurements, have been evaluated by the Owen, Wendt, Rabel and Kaelble (OWRK) method [21]. Both disperse and polar components of SE have been estimated.

Dynamic light scattering (DLS) analysis has been performed at *T* = 25 °C by means of a Zetasizer Nano (Malvern Instruments). DLS measurements have been collected over two weeks.

Atomic force microscopy (AFM) images of the fabricated devices (substrates and deposited graphene) have been taken by means of an XE100 Park instrument operating in non-contact mode (amplitude modulation, silicon nitride cantilever from Nanosensor) at room temperature and under ambient conditions.

### Sensor-device fabrication

Different substrates, paper (Epson, premium glossy photo paper), alumina (purity 96%, Rockwell hardness = 82, resistivity > 1014 Ω·cm, roughness = 12–20 microinches) and Si/SiO_2_ (thermal oxide 300 nm, Silicon Valley), with gold electrodes have been employed as transducers. For all the substrates, interdigitated Cr/Au (30 nm/120 nm) electrodes have been realized by e-beam evaporation (chamber pressure at about 10^−7^ mbar) through a shadow mask.

The inkjet equipment has been a piezoelectric drop-on-demand Dimatix materials printer 2831 (DMP2831) of FUJIFILM USA suitable for the print of functional inks onto flexible and rigid substrates through multi-nozzles printheads.

The graphene-based ink has been printed by keeping the electrode/substrate system at 30 °C and by employing 4 nozzles with a drop space of 25 μm. The chosen pattern of the printed sensing material is a rectangular surface of 9 × 1.75 mm^2^.

The chemiresistors have been prepared by inkjet printing the graphene dispersion onto the pre-patterned substrates and electrically characterized just after the fabrication. The devices have been manufactured with a different number of printed sensing layers depending on the substrates. The number of overlapped printed layers and the base conductance have been taken into account as parameters to address the device-to-device variation. All devices, after printing, have undergone a thermal treatment on a hot plate at 100 °C for 15 min for removing the residual solvent.

### Sensing analysis

Tests for sensing measurements upon NO_2_ have been performed in the gas sensor characterization system (GSCS, Kenosistec equipment) equipped with a stainless steel chamber placed in a thermostatic box, keeping constant the temperature (*T* = 22 °C), the relative humidity (RH = 50%) and under controlled nitrogen environment at atmospheric pressure, by keeping the flow constant at 500 sccm.

All devices, biased at 1 V, have been analyzed through a measurement protocol consisting of an exposure towards the analyte for 10 min, preceded and followed by 20 min long phases in inert atmosphere (baseline and recovery phases, respectively).

## Supporting Information

File 1 Additional experimental data.
