# Supplementary material for: Study of the correlation between sensing performance and surface morphology of inkjet-printed aqueous graphene-based chemiresistors for NO2 detection
Source: Beilstein J Nanotechnol. 2017 May 9;8:1023–31. doi: 10.3762/bjnano.8.103 (PMC5433170; doi:10.3762/bjnano.8.103)
Supplement: File 1 — Additional experimental data. [file Beilstein_J_Nanotechnol-08-1023-s001.pdf]

Supporting Information

for

Study of the correlation between sensing performance and surface morphology of inkjet-printed aqueous graphene-based chemiresistors for NO_2 detection

F. Villani[1], C. Schiattarella[2], T. Polichetti*[1], R. Di Capua[2,3], F. Loffredo[1], B. Alfano[1], M. L. Miglietta[1], E. Massera[1], L. Verdoliva[1] and G. Di Francia[1]

Address: [1]ENEA - R.C. Portici, Piazzale E. Fermi 1, I-80055, Portici (Naples), Italy; [2]Dipartimento di Fisica "E. Pancini", Università di Napoli "Federico II", Via Cintia, I-80126, Naples, Italy and [3]CNR-SPIN UOS Napoli, Via Cintia, I-80126, Naples, Italy

Email: Tiziana Polichetti* - tiziana.polichetti@enea.it

* Corresponding author

Additional experimental data

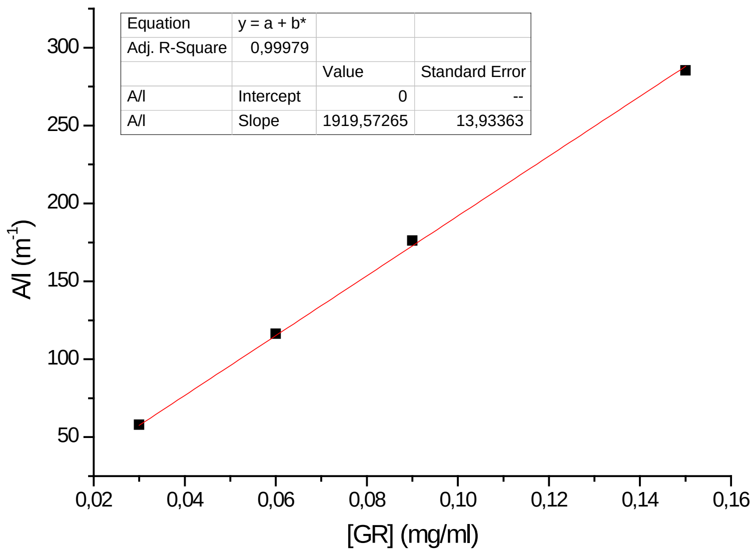

Figure S1: Absorbance per unit length (λ = 660 nm) as a function of the graphite concentration (after centrifugation).

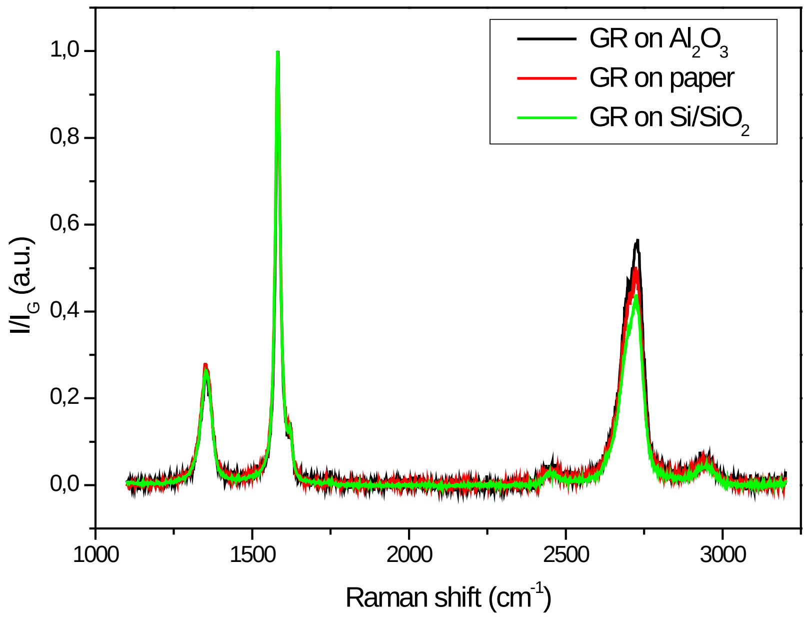

Figure S2: Raman analysis performed on the devices printed on different substrates.

Raman analysis performed on all the devices shows features perfectly overlapped, without any displacement of peaks positions. This is a clear indication of the absence of a doping

effect induced by the substrates. As can be observed, even the $I(D)/I(G)$ ratio is the same for all the sensing films. This demonstrates that the defect density is retained regardless of the substrate.
